# Beclomethasone dipropionate and formoterol fumarate synergistically interact in hyperresponsive medium bronchi and small airways

**DOI:** 10.1186/s12931-018-0770-7

**Published:** 2018-04-12

**Authors:** Luigino Calzetta, Maria Gabriella Matera, Francesco Facciolo, Mario Cazzola, Paola Rogliani

**Affiliations:** 1 Unit of Respiratory Medicine, Department of Experimental Medicine and Surgery, University of Rome “Tor Vergata”, Via Montpellier 1, 00133 Rome, Italy; 2 Unit of Pharmacology, Department of Experimental Medicine, University of Campania Luigi Vanvitelli, Naples, Italy; 3 Thoracic Surgery Unit, “Regina Elena” National Cancer Institute, Rome, Italy

**Keywords:** ICS/LABA FDC, Asthma, Interaction, Medium bronchi, Small airways

## Abstract

**Background:**

Corticosteroids increase the expression of β_2_-adrenoceptors (β_2_-ARs) and protect them against down-regulation. Conversely, β_2_-AR agonists improve the anti-inflammatory action of corticosteroids. Nevertheless, it is still uncertain whether adding a long-acting β_2_-AR agonist (LABA) to an inhaled corticosteroid (ICS) results in an additive effect, or there is true synergy.

Therefore, the aim of this study was to pharmacologically characterize the interaction between the ICS beclomethasone diproprionate (BDP) and the LABA formoterol fumarate (FF) in a validated human ex vivo model of bronchial asthma.

**Methods:**

Human medium and small airways were stimulated by histamine and treated with different concentrations of BDP and FF, administered alone and in combination at concentration-ratio reproducing ex vivo that of the currently available fixed-dose combination (FDC; BDP/FF 100:6 combination-ratio). Experiments were performed in non-sensitized (NS) and passively sensitized (PS) airways. The pharmacological interaction was assessed by using Bliss Independence and Unified Theory equations.

**Results:**

BDP/FF synergistically increased the overall bronchorelaxation in NS and PS airways (+ 15.15% ± 4.02%; *P* < 0.05 vs. additive effect). At low-to-medium concentrations the synergistic interaction was greater in PS than in NS bronchioles (+ 16.68% ± 3.02% and + 7.27% ± 3.05%, respectively). In PS small airways a very strong synergistic interaction (Combination Index: 0.08; + 20.04% ± 2.18% vs. additive effect) was detected for the total concentrations of BDP/FF combination corresponding to 10.6 ng/ml.

**Conclusion:**

BDP/FF combination synergistically relaxed human bronchi; the extent of such an interaction was very strong at low-to-medium concentrations in PS small airways.

**Trial registration:**

Not applicable.

**Electronic supplementary material:**

The online version of this article (10.1186/s12931-018-0770-7) contains supplementary material, which is available to authorized users.

## Background

Combining an inhaled corticosteroid (ICS) with a long-acting β_2_-adrenoceptor (β_2_-AR) agonist (LABA) is the cornerstone for the treatment of adult patients with asthma symptoms when a medium dose of ICS alone fails to achieve control of asthma [1].The scientific rationale for inhaled combination therapy with β_2_-AR agonists and corticosteroids has been debated for a long time [2]. Already in the early 2000s, it was widely recognized that the addition of a LABA to an ICS provides the optimal control of asthma in most patients, and ICS/LABA fixed-dose combinations (FDCs) represent effective controllers in patients with persistent asthma [3]. There is clear evidence that ICS/LABA FDC is superior to either drug given as monotherapy in the clinical management of moderate to severe asthma [1, 4].

Previous works elucidated some mechanisms behind the additive ant-inflammatory effect of adding a LABA to a corticosteroid in the treatment of asthma [5–9]. Nevertheless, to date there is still a large gap in the knowledge on how these two agents delivered in combination lead to superior clinical efficacy. In this regard, recent advances in multiscale modelling by using quasi-3D model may provide the opportunity of investigating how ICSs and LABAs interact each other with respect to their absorption, transport and retention into the lung at the level of lining liquid, epithelium, interstitium, airway smooth muscle (ASM), immune cells, and endothelium [10].

Overall, β_2_-AR agonists reduce the contractile tone of ASM, prevent plasma exudation, and inhibit the release of mediators from inflammatory cells and activation of sensory nerves. Conversely, corticosteroids reduce chronic inflammation and bronchial hyperresponsiveness (BHR) [3]. Taken together, these effects allow achieving an adequate asthma control. However, the intimate interaction between the activation of membrane β_2_-AR and intracellular glucocorticoid receptor (GR) is complex and not fully understood. Unquestionably, corticosteroids enhance the expression of β_2_-AR and protect these receptors against down-regulation in response to chronic activation at the level of ASM cells, whereas β_2_-AR agonists may increase the anti-inflammatory effects of corticosteroids at the level of inflammatory cells [11].

Although each class of drug enhances beneficial actions induced by the other class, the doubts raised by Barnes and Giembycz [3, 4] more than a decade ago on whether adding a LABA to an ICS results in an additive effect, or there is true synergy, are still current. In fact, little information is available on the real pharmacological characterization of ICS/LABA combination [4].

Indeed, the concept of synergy is appealing and extensively used, as confirmed by the last update of the understanding how LABAs enhance the clinical efficacy of ICS in asthma [12]. The increasing recognition that asthma and chronic obstructive pulmonary disease (COPD) are heterogeneous disorders has lead the attention to the needs of a group of patients with clinical features of both asthma and COPD, the so called asthma–COPD overlap syndrome (ACOS) [13]. Nevertheless, to date it is widely recognized that it is premature to recommend the designation of ACOS as a disease entity in both primary and specialist care [14]. In fact, more research is needed to adequately characterize patients and to obtain a validated definition of ACOS that would be based on markers that best predict treatment response in individual patients [15]. Thus, in agreement with the current international recommendations for the diagnosis and treatment of asthma and COPD [1, 16], ICS/LABA FDCs remain the cornerstone therapy for most asthmatic patients rather then COPD patients.

Nevertheless, to date only two studies [5, 17] have assessed the interaction between a β_2_-AR agonist and a corticosteroid by applying correct pharmacological models. These original researches [5, 17] were carried out in murine models of allergic lung inflammation, and not in human airways. While the synergism between bronchodilator agents has been confirmed in clinical studies [18, 19] by using specific pharmacological modeling of drug interaction, to date no clinical trials aimed to assess the potential clinical synergy between ICSs and LABAs have been carried out by using these models, namely the Bliss Independence equation and the Unified Theory [20].

Therefore, the aim of this study was to pharmacologically characterize the impact of beclomethasone dipropionate (BDP) on the bronchorelaxant effect of formoterol fumarate (FF), administered at the concentration-ratio delivered by the currently available FDC in the marked, in human medium bronchi and small airways by using a validated ex vivo model of bronchial asthma.

## Methods

### Tissue collection and preparation

Regions of macroscopically normal lungs were taken from uninvolved areas resected from 16 patients undergoing lobectomy surgery for lung cancer, but without a history of chronic airway disease. Detailed demographic and metric characteristics of patients are reported in Table 1.

**Table 1:** Demographic characteristics of human subjects and normal ranges in agreement with GOLD and GINA [1, 16]

Characteristics	Value	Normal range
Gender (male/female)	8/8	/
Age (years)	50.0 ± 3.0	/
Height (cm)	164.8 ± 2.0	/
Weight (Kg)	68.3 ± 3.0	/
Smoking status:		
current	4	/
former	5	/
never	7	/
IgE (IU/ml)	55.8 ± 5.7	< 100
Pack years	24.4 ± 5.6	/
FEV_1_ (L)	2.71 ± 0.28	/
FEV_1_ (% predicted)	93.1 ± 4.1	> 80
FEV_1_ reversibility (%)	4.8 ± 1.3	< 12%
FVC (L)	3.34 ± 0.36	/
FEV_1_/FVC	0.81 ± 0.01	> 0.7

*FEV*_*1*_ forced expiratory volume in 1 s, *FVC* forced vital capacity, *GINA* Global Initiative for Asthma, *GOLD* Global Initiative for Chronic Obstructive Lung Disease, *IgE* immunoglobulin E, *IU* international units

Tissues were placed in Krebs-Henseleit buffer solution (KH) as previously described [21–23] and transported to the Laboratory of Respiratory Clinical Pharmacology at the University of Rome Tor Vergata (Italy) from a nearby hospital. None of the patients had been chronically treated with inhaled bronchodilators or glucocorticosteroids. Serum immunoglobulin E (IgE) levels determined on the day of surgery were in the normal range (< 100 IU/ml) [24]. Preoperative lung function parameters were generally normal and there were no signs of respiratory infections.

In the laboratory, the airways were cut into rings (sub-segmental bronchi: thickness 1–2 mm, diameter 4–6 mm) and transferred into a 10-ml High Tech 8 Channels Manual Compact Organ Bath system (Panlab Harvard Apparatus, Spain) containing KH buffer solution (37 °C) and aerated with O_2_/CO_2_ (95:5%). Tissues were allowed to equilibrate and the KH buffer solution was constantly changed.

Airways, which were studied in videomorphometry, were cut into precision cut lung slices (PCLS) (bronchioles: thickness < 500 μm, diameter < 1 mm) by a Motorised Advance Vibroslice equipped with ceramic blades (Campden Instruments, UK). Slices were processed without the complications related to the use of confounding agarose gel to inflate the lung or complex parenchymal sections that have numerous contracting elements [25–27]. PCLSs were then mounted into a Visual Imaging and Patching Chamber connected to a Proportional Integral Derivative Temperature Controller with dual thermistor feedback CI7800 (Campden Instruments, UK), containing KH buffer solution (37 °C) and continuously aerated with O_2_/CO_2_ (95:5%).

### Passive sensitization

Isolated airways were rotated overnight at room temperature in tubes containing KH buffer solution in the presence of 10% vol^− 1^ sensitizing serum (passively sensitized bronchi) or 10% vol^− 1^ non-sensitizing serum collected from non-atopic donors (non-sensitized bronchi). Sera were prepared by centrifugation from the whole blood of patients suffering from atopic asthma (total IgE 1000 U ml^− 1^ specific against common aeroallergens) during exacerbation and non-atopic subjects [28, 29], providing signed consent for serum donation. Sera were frozen at − 80 °C in 250 μl aliquots until required. The next morning bronchial tissues were transferred into the isolated organ bath system or videomorphometry chamber containing KH buffer solution (37 °C) and continuously gassed with O_2_/CO_2_ (95:5%).

The passive sensitization is a model that closely mimics important functional characteristics of non-specific bronchial hyperresponsiveness (BHR) in asthmatic patients, as previously reported [28–32].

### Preparation of drugs

The following drugs were used: acetylcholine, beclomethasone dipropionate (BDP), formoterol fumarate (FF), histamine, papaverine. All compounds were obtained by Sigma-Aldrich (Milan, Italy). All products were dissolved in adequate diluents such as distilled water, ethanol and dimethyl sulfoxide (DMSO). The maximum amount of ethanol and DMSO did not influence isolated tissue response [33, 34]. Compounds were stored in small aliquots at − 80 °C until their use.

### Contraction measurement

#### Isolated bronchi

Bronchial rings were connected to isometric force transducers Fort25 (WPI, UK). PowerLab 8/36 and Octal Bridge Amp system (ADInstruments, UK), recorded and analyzed with the LabChart 7 interface software (ADInstruments, UK). Tissues were mounted on hooks, and attached with thread to a stationary rod and the other tied with thread to an isometric force displacement transducer. Airways were allowed to equilibrate by flushing with fresh KH buffer solution. Passive tension was determined by gentle stretching of tissue (0.5–1.0 g) during equilibration. The isometric change in tension was measured by the transducer and the tissue vitality assessed by transmural stimulation (also called electrical field stimulation, EFS) at 25 Hz; when the passive contractile tone reached the plateau, rings were washed three times with KH buffer solution and allowed to equilibrate for 45 min [35, 36].

#### Videomorphometry

Bronchial contraction was evaluated by a stereo microscope Zenith SZR-10 and a digital Optikam-B5 managed by OptikaView7 software (Optika Microscopes, Italy). Airways were allowed to equilibrate by flushing with fresh KH buffer solution until the luminal area was stable. The area into the lumen was measured by the Image Processing and Analysis software ImageJ (http://rsbweb.nih.gov/ij/index.html). The tissue vitality was assessed by acetylcholine (0.3 μM) in order to produce an area reduction of at the least 25% [25, 37–41]. After that, rings were washed three times with KH buffer solution and allowed to equilibrate for 45 min.

### Study design

Following equilibration of non-sensitized and passively sensitized tissues, bronchial rings/slices were sub-maximally contracted by histamine (70% of maximal contraction, EC_70_). After the plateau was reached, semi-logarithmic concentration-response curves (CRCs) to FF were constructed. Each CRC was obtained by the cumulative addition of the drug at intervals of 5–15 min to reach a stable level of relaxation before the next dose administration.

In order to investigate the pharmacological interaction between BDP and FF in the ex vivo model of asthma, non-sensitized and passively sensitized airways were also treated overnight with different concentrations of BDP. After that, the CRCs to FF were constructed on the sub-maximal contractile tone induced by histamine, as described above. BDP and FF were tested at a ratio of concentrations (100:6 weight/weight combination-ratio) reproducing that of the FDC currently approved for the treatment of adult asthmatic patients (BDP/FF 100:6 combination-ratio) [42]. Thus, the range of concentrations of BDP to be combined with FF were chosen in agreement with the concentrations of FF that elicited a detectable bronchorelaxant response.

In the control groups, cumulative concentrations of vehicle was administered and used as time control. At the end of the experiments, papaverine (100 μM) was added to the baths to determine the maximal relaxant response achievable for each isolated airway. These experiments were carried out in both isolated organ bath and PCLS systems, in order to evaluate the relaxant response associated with the ASM strength and related to the increasing of intra-luminal bronchial area [43, 44].

### Analysis

#### Pharmacological analysis

The contractile relaxation of isolated bronchial rings/slices was expressed as a percentage of the maximal relaxation (E_max_, strength/luminal area) induced by papaverine (100 μM) on the submaximal contractile plateau induced by histamine (70% maximal contractility). Appropriate curve-fitting to a sigmoidal models was used to calculate the effect, the maximal response, the concentration inducing 50% maximal effect and the dose inducing 70% maximal effect (E, E_max_, EC_50_ and EC_70_ respectively). The equation used was: log (agonist) vs. response, Variable slope, expressed as Y=Bottom+(Top-Bottom)/{1 + 10^[(LogEC50-X)*HillSlope]}. For the statistical analysis of the potency, pEC_50_ values were used, where pEC_50_ = -LogEC_50_) [45]. If necessary, bell-shaped curves were constructed by fitting models of biological data using nonlinear regressions [45]. For every seven bronchial rings mounted in the isolated organ bath system, one was used as a time control [46].

#### Interaction analysis

The interaction between BDP and FF was tested by applying the Bliss Independence (BI) theory, one of the most commonly used models to study combined effects of substances in vivo, ex vivo and in vitro. This method provides results on the statistical significance of the difference between the expected additive response and the observed effect in CRCs of drug combinations [47–50]. The main assumption of the BI theory is that two or more agents act independently from one another. In particular, if fulfilling the criterion, the mode, and possibly also the site of action of the compounds in the mixture, always differ. The BI theory for two agents is expressed by the following equation “E(x,y) = Ex+Ey-(Ex*Ey)”, where E is the fractional effect, and x and y are the concentrations of compounds in a combination experiment [51]. If the combination effect is higher than the expected value from the above equations, the interaction is synergistic, while if this effect is lower, the interaction is antagonistic. Otherwise, the effect is additive and there is no interaction [51–57]. In this protocol, the BI equation was used to investigate the interaction between BDP and FF administered in combination.

In addition to BI method, also the Unified Theory analysis was carried out, as proposed by Chou and colleagues [49, 58], in order to adequately quantify the magnitude of synergism through the Combination Index outcome and to produce results that are easy to be interpreted through the isobologram representation of data. The Unified Theory is represented by the Median-Effect equation that includes four major biochemical and biophysical equations (Henderson-Hasselbalch, Michaelis-Menten, Hill, and Scatchard), leading to the Combination Index theorem and to the isobologram equation for multiple drug combinations. Thus, the Combination Index is effect-oriented and quantifies the synergism or antagonism, where values < 1, =1, and > 1 indicate synergism, additive effect and antagonism, respectively, whereas the isobologram is concentration-oriented and it is expressed as a graph with equipotency sum of the doses [57, 58].

#### Statistical analysis

Values are presented as mean ± SEM of *n* = 5 bronchi from different subjects. The statistical significance was assessed by the t-test and analysis of variance (ANOVA), where necessary. The level of statistical significance was defined as *P* < 0.05. All data analysis was performed using computer software GraphPad Prism 5 (La Jolla, CA, USA) and CompuSyn (Paramus, NJ. USA).

## Results

### Baseline characteristics of isolated airways

The baseline characteristics of non-sensitized and passively sensitized isolated airways used in this study are reported in Additional file 1.

### Pharmacological characteristics of BDP and FF administered as monocomponents on the histaminergic contractility

FF completely relaxed both non-sensitized and passively sensitized medium bronchi submaximally pre-contracted by histamine, although the potency of FF was significantly (*P* < 0.001) greater in passively sensitized than in non-sensitized bronchi. FF partially relaxed non-sensitized PCLS and completely relaxed passively sensitized PCLS submaximally pre-contracted by histamine. The potency of FF was significantly (*P* < 0.05) greater in passively sensitized than in non-sensitized PCLS. Overall, FF was ≃1.25 logarithm more potent in medium bronchi than in small airways.

After overnight incubation, BDP induced a very weak and not significant (*P* > 0.05 vs. plateau induced by histamine) reduction of the histaminergic contractile tone of both medium bronchi and PCLS, in both non-sensitized and passively sensitized tissues.

Detailed pharmacological characteristics of BDP and FF are reported in Table 2 and specific results of each CRC are shown in Additional file 1: Fig. S1.

**Table 2:** Pharmacological characteristics of BDP and FF administered as monocomponents on the histaminergic contractile tone of medium bronchi and small airways (PCLS) in non-sensitized and passively sensitized tissues

	BDP				FF			
	Medium bronchi		Bronchioles		Medium bronchi		Bronchioles	
	E_max_	pEC_50_	E_max_	pEC_50_	E_max_	pEC_50_	E_max_	pEC_50_
Non-sensitized	< 20%	NC	< 20%	NC	97.00 ± 2.77%	8.26 ± 0.07	72.03% ± 4.34^#^	7.13 ± 0.11^#^
Passively sensitized	< 20%	NC	< 20%	NC	94.96 ± 1.22%	8.89 ± 0.04***	105.30%***	7.52 ± 0.09*^#^

*P < 0.05 and ***P < 0.001 vs. non-sensitized airways of equal diameter, ^#^*P* < 0.001 vs. medium bronchi undergoing the same treatment (statistical significance assessed by t-test). *BDP* Beclomethasone dipropionate, *EC*_50_ The concentration inducing 50% E_max_, *E*_*max*_ maximal effect, *FF* Formoterol fumarate, *NC* Not calculable, *PCLS* Precision cut lung slices, *pEC*_*50*_ -LogEC_50_

### Pharmacological interaction between BDP and FF in non-sensitized and passively sensitized medium isolated bronchi

**Fig. 1:**
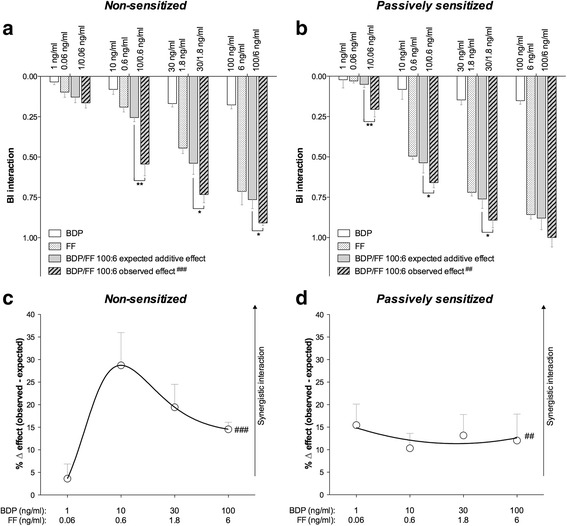
Bliss Independence analysis (**a** and **b**) and delta effect between the observed and expected relaxant response (**c** and **d**) induced by BDP plus FF administered at 100:6 combination-ratio in non-sensitized (**a** and **c**) and passively sensitized (**b** and **d**) human medium bronchi submaximally contracted by histamine. *P < 0.05 and **P < 0.01 vs. the expected additive relaxant effect as predicted by BI equation (statistical significance assessed by Student’s *t* test); ^##^*P* < 0.01 and ^###^P < 0.001 vs. the expected additive relaxant effect as predicted by BI equation (statistical significance assessed by two-way ANOVA). Points represent the mean ± SEM of n = 5 sub-segmental bronchi from different subjects. BI: Bliss independence; BDP: beclomethasone dipropionate; FF: formoterol fumarate

The observed effect induced by increasing concentrations of BDP plus FF combined at 100:6 combination-ratio in both non-sensitized and passively sensitized bronchi pre-contracted by histamine was significantly (*P* < 0.01 determined by two-way ANOVA) greater than the expected additive effect resulting from the BI model (Fig. 1a and b). The maximal synergistic interaction was detected for the drug mixture corresponding to BDP/FF 10/0.6 ng/ml in non-sensitized airways (percentage change: + 28.73% ± 7.25% vs. additive effect; *P* < 0.001), whereas in passively sensitized tissues the extent of synergistic interaction remained constant for BDP/FF combinations administered from 1/0.06 ng/ml to 100/6 ng/ml (overall percentage change: + 12.74% ± 4.62% vs. additive effect; *P* < 0.01) (Fig. 1c and d).

**Fig. 2:**
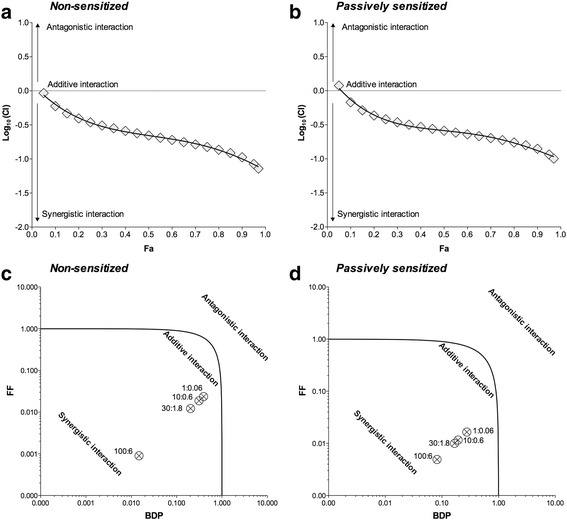
Graphical representation of Unified Theory analysis for BDP/FF combination administered at 100:6 concentration-ratio reporting the logarithmic Combination Index plot (**a** and **b**) and the normalized isobologram (**c** and **d**) in non-sensitized (**a** and **c**) and passively sensitized (**b** and **d**) human medium bronchi submaximally contracted by histamine. Points represent the mean ± SEM of *n* = 5 sub-segmental bronchi from different subjects; the labels of points report the weight/weight ratio (ng/ng) between BDP and FF. BDP: beclomethasone dipropionate; CI: Combination Index; Fa: fraction affected; FF: formoterol fumarate. Fu: fraction unaffected

Synergism was confirmed by the Unified Theory analysis. The logarithmic Combination Index plot showed that the effect of BDP/FF 100:6 combination-ratio was substantially in the area of synergistic interaction (Log_10_ of Combination Index < 0) when the drugs mixtures were tested in both non-sensitized and passively sensitized bronchi (Fig. 2a and b). The isobologram analysis reported that BDP/FF 100:6 combination-ratio was a balanced combination inducing synergistic effect from low to high concentrations, in both non-sensitized and passively sensitized bronchi (Fig. 2c and d).

### Pharmacological interaction between BDP and FF in non-sensitized and passively sensitized small airways

**Fig. 3:**
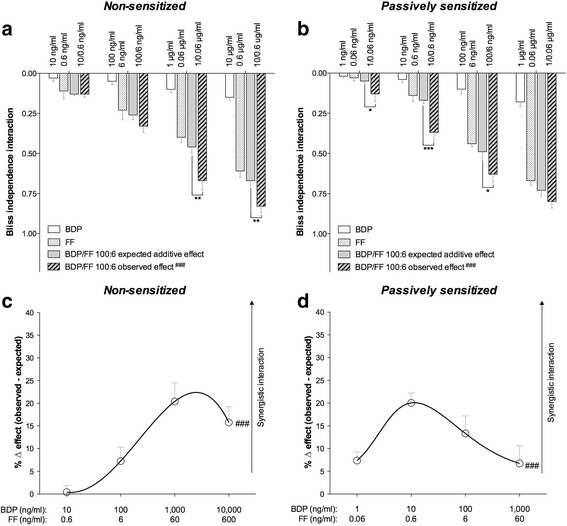
Bliss Independence analysis (**a** and **b**) and delta effect between the observed and expected relaxant response (**c** and **d**) induced by BDP plus FF administered at 100:6 combination-ratio in non-sensitized (**a** and **c**) and passively sensitized (**b** and **d**) human small airways (PCLS) submaximally contracted by histamine. *P < 0.05, **P < 0.01 and ***P < 0.001 vs. the expected additive relaxant effect as predicted by BI equation (statistical significance assessed by Student’s *t* test); ^###^P < 0.001 vs. the expected additive relaxant effect as predicted by BI equation (statistical significance assessed by two-way ANOVA). Points represent the mean ± SEM of n = 5 bronchioles from different subjects. BI: Bliss independence; BDP: beclomethasone dipropionate; FF: formoterol fumarate; PCLS: precision cut lung slices

Combining increasing concentrations of BDP plus FF elicited a significant (*P* < 0.001 determined by two-way ANOVA) synergistic relaxant effect of both non-sensitized and passively sensitized bronchioles pre-contracted by histamine (Fig. 3a and b). The maximal synergistic interaction was detected for BDP/FF combined at 1/0.06 μg/ml in non-sensitized small airways (+ 20.41% ± 4.10% vs. additive effect; P < 0.001), and for BDP/FF combined at 10/0.6 ng/ml in passively sensitized bronchioles (+ 20.04% ± 2.18% vs. additive effect; *P* < 0.01) (Fig. 3c and d).

**Fig. 4:**
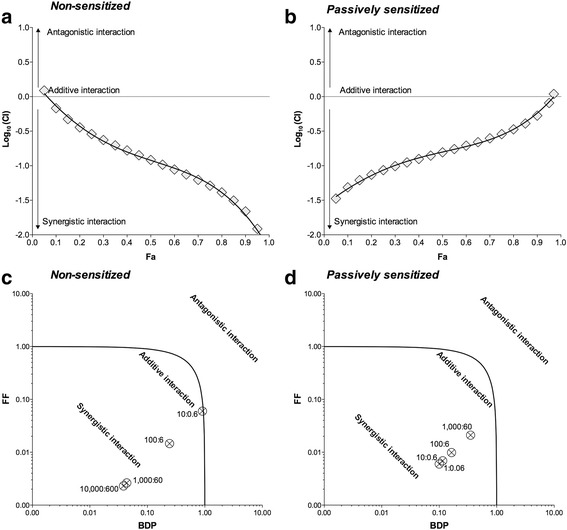
Graphical representation of Unified Theory analysis for BDP/FF combination administered at 100:6 concentration-ratio reporting the logarithmic Combination Index plot (**a** and **b**) and the normalized isobologram (**c** and **d**) in non-sensitized (**a** and **c**) and passively sensitized (**b** and **d**) human small airways (PCLS) submaximally contracted by histamine. Points represent the mean ± SEM of n = 5 bronchioles from different subjects; the labels of points report the weight/weight ratio (ng/ng) between BDP and FF. BDP: beclomethasone dipropionate; CI: Combination Index; Fa: fraction affected; FF: formoterol fumarate. Fu: fraction unaffected; PCLS: precision cut lung slices

The Unified Theory approach confirmed the synergy between BDP and FF at the level of small airways. The logarithmic Combination Index plot indicated that the synergy of BDP/FF 100:6 combination-ratio was substantially greater at higher concentrations than at lower concentrations in non-sensitized PCLS (Fig. 4a). On the other hand, in passively sensitized small airways we detected a reverse trend, characterized by a greater synergistic interaction at lower concentrations compared with that at higher concentrations (Fig. 4b). Similarly, the isobologram analysis reported that the extent of the synergistic interaction between BDP and FF was directly related with the concentrations of drugs mixture in non-sensitized bronchioles (Fig. 4c). On the other hand, the extent of interaction was inversely related to the concentrations of BDP/FF combinations in passively sensitized small airways (Fig. 4d).

### Magnitude of synergistic interaction in medium and small airways

Overall, BDP/FF combination elicited synergistic relaxant response of ASM submaximally contracted by histamine.

In medium bronchi synergism (overall Combination Index: 0.425) was detected at low concentrations inducing ≤25% E_max_, and strong synergism (overall Combination Index: 0.178) from concentrations eliciting ≥50% E_max_.

In non-sensitized small airways the extent of synergism was strong (overall Combination Index: 0.205) at low concentrations inducing from 25% to 50% E_max_, and very strong (overall Combination Index: 0.035) at higher concentrations.

Very strong synergistic interaction (overall Combination Index: 0.070) was detected in passively sensitized small airways yet at very low concentrations inducing 15–25% E_max_, and the extent of interaction remained strong (overall Combination Index: 0.215) up to concentrations eliciting submaximal bronchorelaxant effect (75% E_max_).

Table 3 summarizes the magnitude of the pharmacological interaction between BDP and FF administered at 100:6 combination-ratio in non-sensitized and passively sensitized human medium and small airways.

**Table 3:** Magnitude of the pharmacological interaction between BDP and FF administered at 100:6 combination-ratio in non-sensitized and passively sensitized human medium and small airways (PCLS)

	Medium bronchi				Small airways			
	Non-sensitized		Passively sensitized		Non-sensitized		Passively sensitized	
Relaxant effect (% E_max_)	Combination Index	Interaction magnitude	Combination Index	Interaction magnitude	Combination Index	Interaction magnitude	Combination Index	Interaction magnitude
15	0.47	+++	0.51	+++	0.47	+++	0.06	+++++
25	0.34	+++	0.38	+++	0.29	++++	0.08	+++++
50	0.22	++++	0.26	++++	0.12	++++	0.15	++++
75	0.15	++++	0.20	++++	0.05	+++++	0.28	++++
90	0.10	++++	0.14	++++	0.02	+++++	0.52	+++

The smaller the Combination Index, the greater the synergistic interaction. +++: synergism; ++++: strong synergism; +++++: very strong synergism. *BDP* Beclomethasone dipropionate, *FF* formoterol fumarate, *PCLS* Precision cut lung slices

## Discussion

The results of this study confirmed that FF is a potent and effective bronchorelaxant agent at the level of medium bronchi, as previously reported by studies carried out in 1990s [59, 60]. Although in a previous study we documented that FF is able to abolish the contraction of small airways induced by acetylcholine [47], in the present research FF did not completely relax bronchioles submaximally pre-contracted by histamine. However, we have provided for the first time the evidence that FF is ≃0.5 logarithm more potent in passively sensitized than in non-sensitized airways and completely abolishes the histaminergic tone of passively sensitized PCLS. These evidences indicate that FF has a beneficial bronchorelaxant impact especially in human hyperresponsive airways. On the contrary, the results indicated that the overnight incubation with BDP did not modulate the bronchial contractility induced by histamine in both medium and small airways, even after passive sensitization procedure.

Conversely, combining BDP with FF at 100:6 concentration-ratio not only improved the effectiveness of the β_2_-AR agonist, but elicited a synergistic bronchorelaxant effect in both medium and small airways, either non-sensitized or passively sensitized.

The BI analysis showed that the in non-sensitized tissues the concentration of drugs mixture necessary to induce the maximal synergistic interaction in bronchioles was ≃2 logarithms higher than that necessary to elicit the greatest synergism in medium bronchi. The total concentration of BDP/FF combination required to induce submaximal relaxation of non-sensitized medium bronchi was in the order of magnitude of ng/ml, whereas in small airways the order of magnitude to elicit the same effect was in the range of μg/ml. On the other hand, in passively sensitized tissues low concentrations of BDP/FF combination, in the order of magnitude of ng/ml, were sufficient to submaximally relax both medium and small airways. These findings prove that passively sensitized small airways are more sensitive to the beneficial synergistic interaction induced by adding BDP to FF, compared to non-sensitized bronchioles.

The BI approach showed that the BDP/FF 100:6 combination-ratio induced statistically significant synergism, and permitted to quantify the overall extent of bronchodilation and compare the observed synergism with the expected additive effect. Nevertheless, the BI equation did not allow assessing what is the real magnitude of synergism occurring between BDP and FF. At concentrations of drugs mixture corresponding to EC_50_ or below it is not difficult to detect synergism, whereas a synergistic interaction may be more difficult to be detected at higher concentrations producing a submaximal effect. Therefore, after have performed the statistical analysis of the synergism between BDP and FF by using the BI model, we have carried out a further evaluation by applying the Unified Theory in order to quantify the magnitude of the interaction [57].

The analysis of the logarithmic Combination Index plots and isobolograms evidenced that in medium bronchi, either non-sensitized or passively sensitized, BDP/FF 100:6 combination-ratio elicited a certain level of bronchorelaxant synergism, even at high concentrations. In non-sensitized small airways low concentrations induced additive effect, and very high concentrations were necessary to elicit synergistic interaction. Surprisingly, in passively sensitized small airways, even very small total concentrations of BDP/FF combination, ranging between 1.06 ng/ml and 10.6 ng/ml, were effective in producing a marked synergistic relaxation of ASM. Certainly the increased acute bronchorelaxant effect of FF in isolated airways incubated overnight with BDP can be prevalently due to the genomic effect of the corticosteroid [61, 62], although investigating this matter was beyond the scope of our study.

The findings of this study are undoubtedly interesting, considering the potential translational implications. In fact, we have previously demonstrated that the relaxant effect of bronchodilator agents detected ex vivo in human medium isolated airways was related with their impact in vivo on the changes in forced expiratory volume in 1 s (FEV _1_) [63]. Furthermore, the luminal area of small airways studied by using PCLS with an internal diameter < 2 mm is related with the flow in small airways that, in turn, seems to be associated with the forced expiratory flow between 25% and 75% of vital capacity (FEF 25–75) [64, 65]. Overall, the flow resistance in medium and small bronchi contributes to the total airway resistance in asthmatic patients, which may influence also the alveolar moiety as demonstrated by the measurement of nitric oxide at different flow rates [66–68].

Lower airways significantly contribute to the severity of chronic obstructive pulmonary disorders, such as asthma and COPD [69]. The dysfunction of bronchioles has been also demonstrated in specific asthma phenotypes, namely nocturnal asthma, exercise-induced asthma, and allergic asthma [70, 71]. Thus, delivering an adequate amount of inhaled ICS/LABA combination to distal airways represents a central target to treat asthmatic patients. However, although the new generation of inhaler devices emitting extrafine formulations have lead to an improved lung deposition of drugs mixture, and a more effective aerosol penetration into the lung periphery [70, 72], in asthmatic patients approximately two-thirds of extrafine formulation of BDP/FF FDC are deposited in the central lung region, and only one-third reaches the peripheral lung [73, 74]. Therefore, it is crucial that the amount of drugs mixture that reaches small airways is effective at concentrations lower than those detectable in larger airways, and that the extent of effectiveness remains sustained also at the low concentrations that are present in the airways immediately before the next dose is inhaled.

In this regard, the very strong synergistic interaction elicited by low concentrations of BDP/FF administered at 100:6 combination-ratio in hyperresponsive small airways may explain the superiority of BDP/FF FDC (400/24 μg daily) delivered via an extrafine formulation in improving asthma control, compared to the combination of the same drugs formulated as larger non-extrafine agents administered at equipotent doses [75]. Furthermore, considering lung functional parameters related with peripheral airway dysfunction, extrafine BDP/FF FDC treatment was superior to an equipotent dose of the non-extrafine fluticasone propionate/salmeterol combination in improving air trapping in moderate to severe asthmatic patients [76]. The beneficial impact of BDP/FF FDC (400/24 μg daily) on small airways in asthma was further confirmed by a pilot study that demonstrated an improvement in closing capacity after 12 weeks of treatment [71]. Intriguingly, in this study [71] the authors also evidenced that BDP/FF FDC was effective in decreasing the BHR of larger airways. This finding indirectly confirms the results of our study, with regard to the evidence that combining an ICS with a LABA can target both medium and small hyperresponsive airways, leading to bronchorelaxant synergistic interaction.

Although the data of the present study result from a widely validated human model of non-specific BHR typical of bronchial asthma [23, 36, 44, 77–80], this research remains an ex vivo study characterized by intrinsic limitations [27, 57]. The findings of ex vivo studies aimed to assess the pharmacological interaction between drugs characterized by different mechanisms of action [81] have been generally confirmed by a translational approach in clinical trials [18, 19], and vice versa [82, 83]. Nevertheless, we must highlight that, although the responses of human isolated ASM strictly resembles those elicited in vivo, results obtained from ex vivo studies need to be confirmed by clinical trials specifically designed to detect pharmacological interaction of drugs mixture [57, 82].

One of the major objectives of having synergistic drug combinations is to reduce the dose of the drugs used, thereby reducing the risk of adverse events, while optimizing the efficacy [57, 58]. In this respect, our data support the delivery of BDP/FF 100:6 combination-ratio via extrafine formulation to reduce the total dose of the monocomponents, improve the distribution in the lung, and optimize the effectiveness, compared to non-extrafine formulations. This approach permits to reduce the deposition of drugs mixture in the oropharynx, resulting in decreased systemic absorption through the gastrointestinal tract and obvious beneficial consequences on the safety profile [84].

A recent and extensive review of Newton and Giembycz [12] attempted to explain how LABAs enhance the clinical efficacy of ICS in asthma. The authors reported that combining an ICS with a LABA might produce profound synergy at the level of genes and proteins expression involved in ASM contractility and airway inflammation [12]. Unfortunately, the original studies [61, 85–88] cited in that review [12] to support the positive interaction between ICSs and LABAs provided arbitrary interpretation of synergy because no methods were used to adequately analyze the real drug interaction. Furthermore, the functional impact of combining a corticosteroid with a β_2_-ARs was indirectly assessed by using cytosolic surrogate of ASM contractility [86]. In any case, even assuming that an ICS/LABA combination can modulate the expression of genes and proteins in asthmatic ASM, it is not implicit that such an interaction may lead to functional synergy [62]. Therefore, at the best of our knowledge, the present study provides for the first time the evidence-based pharmacological characterization of the synergism between an ICS and a LABA, at least with regard to the beneficial impact against BHR in human airways.

## Conclusions

This study indicates that BDP/FF administered at 100:6 combination-ratio induces synergistic bronchorelaxant effect in human medium bronchi and small airways, and that the magnitude of this synergy is greater in hyperresponsive airways than in non-sensitized tissues. Further research is needed to characterize the intimate mechanism/s leading to such an extensive interaction, and assess if combining an ICS with a LABA may lead also to synergistic anti-inflammatory effect in human airways.

## Additional file

Additional file 1: Baseline characteristics of bronchial tissue used in the study and CRCs to BDP and FF. (DOCX 431 kb)

## Abbreviations

ANOVA: analysis of variance
ASM: airway smooth muscle
BDP: beclomethasone dipropionate
BHR: bronchial hyperresponsiveness
BI: Bliss Independence
CRC: concentration-response curves
E: effect
EC_n_: n% E_max_
EC_n_: n% E_max_
E_max_: maximal effect
FDC: fixed-dose combination
FEV_1_: forced expiratory volume in 1 s
FF: formoterol fumarate
FVC: forced vital capacity
GR: glucocorticoid receptor
ICS: inhaled corticosteroid
IgE: immunoglobulin E
KH: Krebs-Henseleit
LABA: long-acting β_2_-AR agonist
PCLS: precision cut lung slices
pEC_50_: potency, -LogEC_50_
β_2_-AR: β_2_-adrenoceptor
