# Supplementary material for: Beclomethasone dipropionate and formoterol fumarate synergistically interact in hyperresponsive medium bronchi and small airways
Source: Respir Res. 2018 Apr 12;19:65. doi: 10.1186/s12931-018-0770-7 (PMC5897944; doi:10.1186/s12931-018-0770-7)
Supplement: Supplementary file 1 — Baseline characteristics of bronchial tissue used in the study and CRCs to BDP and FF. (DOCX 431 kb) [file 12931_2018_770_MOESM1_ESM.docx]

**Beclomethasone dipropionate and formoterol fumarate synergistically interact in hyperresponsive medium bronchi and small airways**

Luigino Calzetta^1^, Maria Gabriella Matera^2^, Francesco Facciolo^3^, Mario Cazzola^1^, Paola Rogliani^1*^

1. Department of Experimental Medicine and Surgery, University of Rome Tor Vergata, Rome, Italy
2. Department of Experimental Medicine, Unit of Pharmacology, Second University of Naples, Naples, Italy.
3. Regina Elena National Cancer Institute, Thoracic Surgery Unit, Rome, Italy.

*Corresponding author: Paola Rogliani, Department of Experimental Medicine and Surgery, Via Montpellier 1, 00133 - Rome, Italy, Tel: +39 06 2090 4656. E-mail address: paola.rogliani@uniroma2.it.

**Results**

**Baseline characteristics of isolated airways**

Bronchial tissue collected from current, former and never smoker patients were equally distributed across the treatments of the study. Independently by the smoking habit of donors, there were no significant differences (P>0.05) between the baseline characteristics of the human isolated bronchial rings employed for the different treatments concerning the wet weight (82.3±3.0 mg) and contractile response induced by EFS 25 Hz (415.4±36.5 mg). Analogously, no significant differences (P>0.05) were detected between the baseline characteristics of the PCLS employed for the different treatments concerning the lumen diameter (0.82±0.04 mm) and contractile response induced by acetylcholine (40.5%±3.8%).

In preliminary experiments, passively sensitized bronchial rings and PCLS showed significant (P<0.001) BHR to histamine when compared with non-sensitized bronchial tissues (overall delta pEC_50_ 1.22±0.04). In non-sensitized bronchial rings and PCLS the EC_70_ to histamine corresponded to 30 µM and 6 µM, respectively. In passively sensitized tissues the EC_70_ to histamine was 0.8 logarithm lower than in non-sensitized airways.


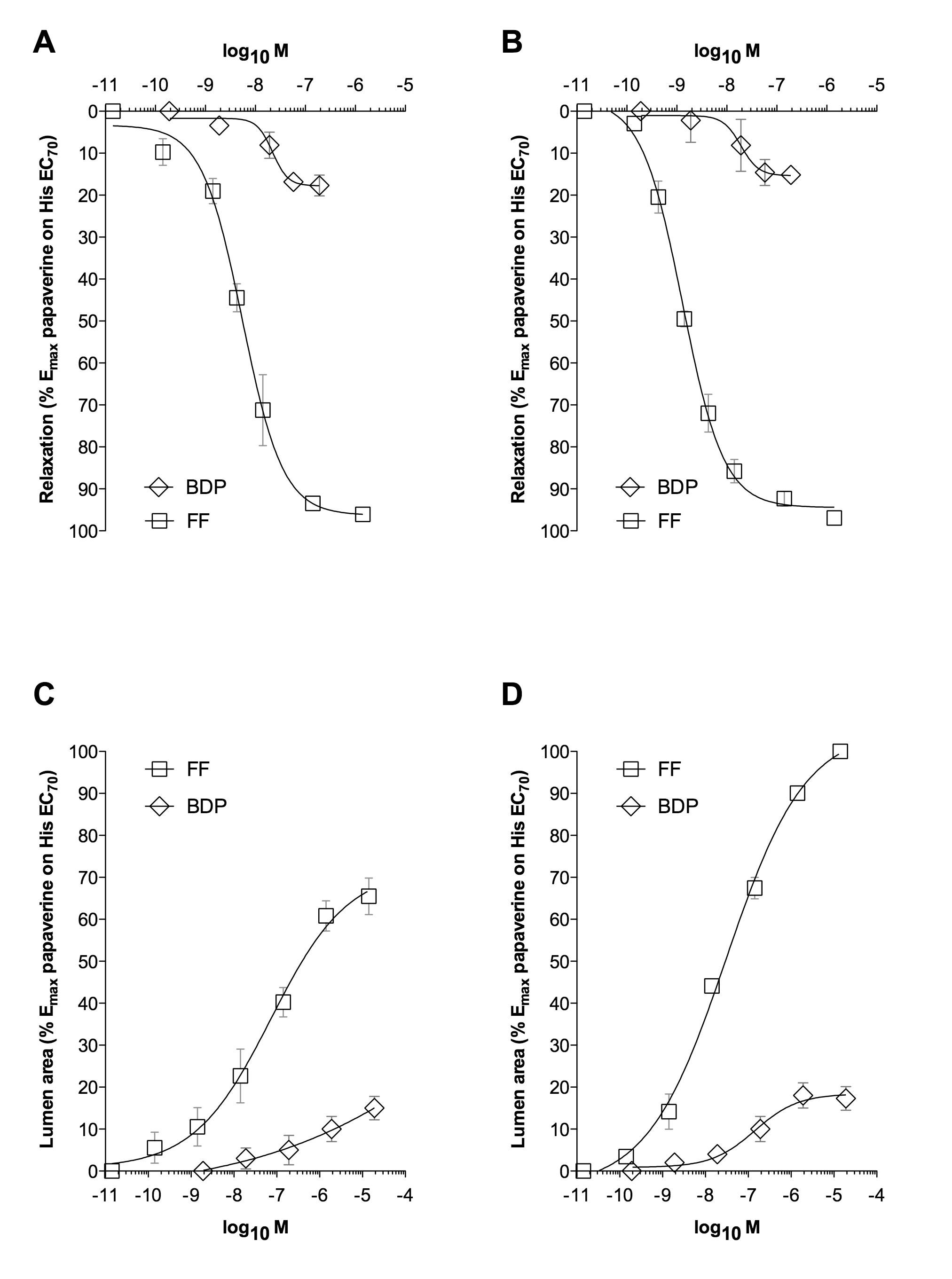


**Figure S1.** Concentration-response curves to formoterol fumarate (FF) and beclomethasone diproprionate (BDP) in human medium isolated bronchi (sub-segmental bronchi: A and B) and human small airways (bronchioles via precision cut lung slices [PCLS]) sub-maximally contracted by histamine (70 % maximal effect: EC_70_). Experiments have been performed in non-sensitized (A and C) and passively sensitized (B and D) bronchial tissue. Points represent the mean±SEM of n=5 bronchial tissue from different subjects.
